# Editorial: Molecular Epidemiology of Fungal Infections

**DOI:** 10.3389/fcimb.2022.939140

**Published:** 2022-05-31

**Authors:** Min Chen, Abdullah M. S. Al-Hatmi, Jianping Xu, G. Sybren De Hoog

**Affiliations:** ^1^Department of Dermatology, Shanghai Key Laboratory of Medical Mycology, Changzheng Hospital, Shanghai, China; ^2^Natural & Medical Sciences Research Center, University of Nizwa, Nizwa, Oman; ^3^Center of Expertise in Mycology, Radboud University Medical Center/Canisius Wilhelmina Hospital, Nijmegen, Netherlands; ^4^Department of Biology, McMaster University, Hamilton, ON, Canada

**Keywords:** genetic divercity, molecular epidemiology, fungal infections, antifungal resistance, fungal pathogen

The term “molecular epidemiology” was first proposed in the 1970s and has since been used to describe any study that uses molecular markers to analyze disease patterns at the population level, including infectious and non-infectious diseases such as SARS-COVID-19 and cancer (Tümmler, 2020). With the rapid development in molecular technology and computer science since the 1990s, molecular epidemiological studies of fungal infections have made tremendous progresses, including studies on plant, animal, and human fungal pathogens (Tarasevich et al., 2003; Tümmler, 2020). Such studies have revealed and refined our understandings of fungal disease outbreaks, transmission dynamics, risk factors, pathogenesis, antifungal resistance, and the genetic and genomic attributes of pathogenic fungi, particularly those that are emerging fungal pathogens. This Special Topic was organized to capture some of the most recent developments and inform the infectious diseases, medical mycology, and public health communities about the progresses and impacts of molecular epidemiology of fungal infections. In the Research Topic on “Molecular Epidemiology of Fungal Infections”, a total of 12 articles have been accepted and published, covering a range of human fungal pathogens as well as different aspects of these pathogens. Interestingly, the majority of the papers (75%; 9/12) were written by Chinese researchers, reflecting the overall trend seen in other journals covering similar topics and suggesting the rapid development of this topic in China.

Over the last 30 years, genotyping methods for studying culturable fungal strains has undergone significant changes, from anonymous fingerprinting techniques such as random amplified polymorphic DNA (RAPD) to sequence-based techniques such as multi-locus sequence typing (MLST) and whole-genome sequencing (Tümmler, 2020). Hong et al., reviewed the diversity of molecular marker techniques that has been used for analyzing the human pathogenic *Cryptococcus* species, specifically members of the *Cryptococcus neoformans* and *Cryptococcus gattii* complexes and how such analyses improved our understanding of this group of fungi. Following a thorough discussion of the advantages and disadvantages of individual molecular markers, the authors proposed that MLST and whole genome sequence typing (WGST) will be the gold standards for continued strain genotyping and epidemiological investigations of the human pathogenic *Cryptococcus* species (Hong et al.). As a model organism for medical fungi, this review summarized not only the evolution of molecular techniques for fungal strain typing but also how those techniques improved our understanding of cryptococcal epidemiology and the evolutionary history of human pathogenic *Cryptococcus*.

Differences in genotype distribution and antifungal resistance have been observed among clinical and environmental isolates of *C. neoformans* in China (Chen et al., 2018; Chen et al., 2021). Yang et al., analyzed 199 clinical isolates of *C. neoformans* from Jiangxi, China, using the MLST consensus scheme and the Clinical and Laboratory Standards Institute (CLSI) M27-A3 method. The study found that ST5/VNI was the most predominant genotype (approximately 89.5%) of clinical *C. neoformans* isolates in the region, along with three novel genotypes (ST656, ST657, and ST658). A large proportion (approximately 43.2%) of the isolates were not sensitive to fluconazole at a MIC_50_ ≥ 8 μg/ml.

Harun et al., investigated 188 global clinical, veterinary, and environmental isolates of *Scedosporium aurantiacum* using a 6-locus MLST scheme. A markedly high genetic diversity with 159 unique sequence types (STs) was observed in this important fungal pathogen in a range of clinical settings. The results of phylogenetics, network and linkage disequilibrium analyses revealed evidence of recombination in the species and suggested that *S. aurantiacum* may have originated within the Australian continent and spread to other regions.

Equine histoplasmosis commonly known as epizootic lymphangitis (EL) is a neglected granulomatous disease of equine that is endemic to Ethiopia, and is caused by the *Histoplasma capsulatum* variety *farciminosum* (Scantlebury et al., 2016). Ameni et al., performed a phylogenetic analysis of 54 veterinary isolates from horses in Ethiopia using the internal transcribed spacer region of rRNA genes (ITS) sequence. The study’s findings indicated that the Ethiopian isolates were closely aggregated with isolates of the South American A and Eurasian clades, but more distantly related to isolates from the North America 1 and 2 clades and the South American B clade. This study highlights the potential origins and transmission routes of Histoplasmosis in Ethiopia.

The *Prototheca* alga is the only chlorophyte capable of causing infections in humans, with *Prototheca wickerhamii* serving as the main causal agent. Guo et al., used nanopore long-read and Illumina short-read technologies to sequence the genomes of two *P. wickerhamii* isolates, S1 and S931, to investigate the evolution of *Prototheca* and the genetic basis for its pathogenicity. The assembled nuclear genome was 17.57 Mb in size with 19 contigs and 17.45 Mb with 26 contigs for isolates S1 and S931, respectively. There were approximately 5,700 predicted protein-coding genes, with over 96% of these genes could be annotated with a gene function. This study provides in-depth insights into the genome sequences of two clinical isolates of *P. wickerhamii* to contribute to the basic understanding of this species.

Chen et al., analyze 110 clinical *Candida* isolates from a tertiary care teaching hospital from Jiangxi, China using RAPD genotyping and the Sensititre ™ YeastOne YO10 panel. *Candida albicans* was the predominant species (approximately 36.3%), followed by *C. parapsilosis* (approximately 33.6%), *C. tropicalis* (approximately 19.1%), *C. glabrata* (approximately 8.2%), *C. rugosa* (approximately 1.8%), and *C. haemulonii* (approximately 0.9%). Using RAPD typing, *C. albicans* isolates could be grouped into five clusters, *C. parapsilosis* and *C. tropicalis* isolates into seven clusters, and *C. glabrata* isolates into only one cluster comprising six strains. Antifungal testing revealed that the isolates had the highest overall resistance against fluconazole (approximately 6.4%), followed by voriconazole (approximately 4.6%). In the azole-resistant isolates, the most common amino acid substitution was 132aa (Y132H, Y132F) within the *erg11* gene. Cluster F had approximately 75% azole-resistant *C. albicans* isolates, while Cluster Y had two azole-resistant *C. tropicalis* isolates.

To date, few studies have reported the incidence of pulmonary co-infection of fungi and bacteria. Zhao et al., used the mNGS technique to analyze 119 patients with fungal infections, 48 (approximately 40.3%) of which had pulmonary fungal and bacterial co-infection. The most commonly identified fungi species were *Aspergillus*, *Pneumocystis*, and *Rhizopus*, and the most commonly identified bacterial species were *Pseudomonas aeruginosa*, *Acinetobacter baumannii*, and *Stenotrophomonas maltophilia*. The results of the study suggest the incidence of fungal and bacterial co-infection in patients with pulmonary fungal infections could not be ignored, which may also guide the use of antibacterial drugs in these patients.

Studies on antifungal susceptibility and resistance mechanisms also are popular Research Topics in molecular epidemiology of fungal infections. Chen et al., used the CLSI M59 guideline and Sensititre YeastOne™ system to analyze 11 *Diutina catenulata* (*Candida catenulata*) isolates collected from the China Hospital Invasive Fungal Surveillance Net (CHIF-NET) Program. The results showed that itraconazole (0.06-0.12 μg/ml), posaconazole (0.06-0.12 μg/ml), amphotericin B (0.25-1μg/ml), and 5-flucytosine (range, 0.06-0.12 μg/ml) had low MICs, whereas echinocandins had high MICs (≥4 μg/ml) in four isolates. Common ERG11 mutations, including F126L/K143R, were found in isolates with high MIC values for azoles. Two frequent amino acid alterations corresponding to high MIC values of echinocandin, F621I (F641) and S625L (S645), were also found in spot 1 region of FKS1. There was also one new amino acid alteration, I1348S (I1368). The high MIC values for various antifungals in the study suggest that the treatment of invasive infections caused by *D. catenulate* may be challenging.

Arastehfar et al., collected 58 *Candida parapsilosis* isolates that recovered from the bloodstream of 47 patients at Turkey’s Ege University Hospital between January 2019 and January 2020. These isolates were genotyped by ITS sequencing and multi-locus microsatellite typing (MLMT). Antifungal susceptibility testing was performed in accordance with the CLSI M60 protocol, and the *ERG11* and HS1/HS2-*FKS1* sequences were sequenced. Results showed that Y132F was the most common mutation in Erg11. All Y132F-positive isolates were found in one large cluster and were mostly recovered from patients admitted to the chest disease and pediatric surgery wards. In Turkey, the study emphasizes the importance of strict infection control strategies, antifungal stewardship, and environmental screening.

Li et al., analyzed the whole genome sequence data from two Chinese *Candida auris* isolates as well as 356 isolates archived in the European Bioinformatics Institute (EBI) databases, which were analyzed by bioinformatics using machine learning classifiers based on Python 3.8.4 software. Two machine learning algorithms, based on the balanced test and imbalanced test, were designed and evaluated. The results obtained using the balanced test set were superior to those obtained using the imbalanced test set for the majority of drugs. This study suggested that machine learning classifiers are a useful and cost-effective method for identifying fungal drug resistance-related mutations, which could be very important for research into the mechanism underlying antifungal resistance observed in *C. auris*, an emerging fungal pathogen.

Liu et al., investigated the clinical and molecular characteristic of 45 chromoblastomycosis (CBM) cases in Guangdong, China, a CBM hotspot. The mean age of the patients was 61.38 ± 11.20 years and the gender ratio (male to female) was 4.6:1. Approximately 29% of the cases had underlying diseases, and a verrucous form was the most common clinical manifestation (approximately 42%). Approximately 62% of the clinical isolates were identified as *F. monophora*, followed by *F. nubica* (approximately 38%). *In vitro* antifungal susceptibility test revealed that azoles (PCZ 0.015_0.25 μg/ml, VCZ 0.015_0.5 μg/ml, and ITZ 0.03_0.5 μg/ml) and TRB (0.015_1 mg/ml) had low MICs. The main therapeutic strategy used for 31 of 45 cases was itraconazole combined with terbinafine, and approximately 68% of them improved or were cured. The findings of this study are likely to represent regional trends in this subtropical hyper-endemic area of CBM, and they will contribute to better CBM management and clinical therapy.

Hu et al., described a rare subcutaneous infection caused by *Dirkmeia churashimaensis* and reviewed previously published human Ustilaginales infections. Over the course of 2 years, an 80-year-old female farmer developed extensive plaques and nodules on her left arm. Pathological and microbiological examinations revealed that this infections was caused by a new pathological agent, *D. churashimaensi*. The patient was successfully cured by oral itraconazole. A literature review suggested amphotericin B, posaconazole, itraconazole, and voriconazole had good activity against these reported strains, whereas fluconazole, 5-flucytosine, and echinocandins usually showed low susceptibility. Itraconazole was effective against subcutaneous infections. The case report and literature review reveal that *D. churashimaensis* can be a fungal opportunist. A proper identification of fungi and antifungal susceptibility tests can be crucial for clinical treatment.

In summary, the published articles in this Research Topic of “Molecular Epidemiology of Fungal Infections” covered a wide range or research, including: (i) the diverse molecular markers used to investigate genetic diversity and antifungal susceptibility of pathogenic fungi; (ii) molecular epidemiologic investigations of fungal outbreaks/epidemics, routes of spread, risk factors, pathogenesis, and environmental distributions, and (iii) applications of next-generation sequencing in molecular epidemiology of fungal infections to addressing new research questions. We hope that this collection will be useful for future research in the field on molecular epidemiology of fungal infections.

## Author Contributions

MC was a guest associate editor of the Research Topic and wrote the paper text. AA-H, JX and GH were guest associate editors of the Research Topic and edited the text. All authors contributed to the article and approved the submitted version.

## Funding

MC is funded in part by the grants from National Natural Science Foundation of China (81720108026 and 82172291). JX is funded in part by grants from Natural Science and Engineering Research Council of Canada (RGPIN-2020-05732) and McMaster University (Global Science Initiative 2020).

## Conflict of Interest

The authors declare that the research was conducted in the absence of any commercial or financial relationships that could be construed as a potential conflict of interest.

## Publisher’s Note

All claims expressed in this article are solely those of the authors and do not necessarily represent those of their affiliated organizations, or those of the publisher, the editors and the reviewers. Any product that may be evaluated in this article, or claim that may be made by its manufacturer, is not guaranteed or endorsed by the publisher.
